# Computational Modeling and Characterization of Nanoporous Films Assembled by Deposition of Au Nanoparticles

**DOI:** 10.3390/nano16110702

**Published:** 2026-06-05

**Authors:** Giacomo Becatti, Francesca Baletto

**Affiliations:** 1 Department of Physics, University of Milan, Via Celoria, 16, I-20133 Milan, Italy; giacomo.becatti@unimi.it; 2 Scientific Computing Center, Karlsruhe Institute of Technology, 76131 Karlshrue, Germany

**Keywords:** molecular dynamics, Au nanoparticles, porous nanofilms, self-assembly

## Abstract

Nanoporous films assembled by low-kinetic-energy deposition of individual nanoparticles are complex nanomaterials for a variety of applications, from gas sensing to neuromorphic computing. We develop a numerical strategy for assembling metallic nanoparticles into 25–40 nm thick films from an arbitrary distribution of Au nanoparticles in terms of their initial size and shape. We characterize the structural properties of the assembled films as a function of the initial nanoparticle distribution. The morphology of the deposited nanoparticles affects nanofilm thickness, porosity, and its internal structure, including the length, type, and density of dislocations. Film porosity and the average dislocation length mainly correlate with the size of deposited nanoparticles. At the same time, thickness and dislocation density can also be affected by the shape of the larger nanoparticles deposited.

## 1. Introduction

Self-organizing techniques, such as self-assembly, are well-established tools for manufacturing nanomaterials. Among them, nanofilms formed by the assembly of individual nanoparticles into films or wires are attracting increasing attention [1,2,3]. After a low-energy deposition, nanoparticles retain their individuality, leading to a complex structure characterized by a dense network of junctions and grain boundaries [4], which makes those nanomaterials potential candidates in a range of applications from catalysis [5] to sensing [6], and to more exotic fields such as components of neuromorphic circuits [7,8,9]. For example, the gas-phase cluster deposition of gold nanoparticles (AuNPs) serves as building blocks for memristors. Nanoassembled AuNPs show non-ohmic electrical behavior and reproducible resistive switching. Those characteristics make them optimal candidates for reservoir computing [10,11,12,13,14], highly requested to satisfy the need of artificial intelligence, which requires faster and faster physical processing units, as brain-like computation can offer.

The fabrication of devices based on AuNP-assembled films requires a deep atomistic understanding of how the individual cluster morphology influences the nanoscale film structure and its properties. There is a profound difference between nanofilms formed from atom deposition or nanoparticle-assembled films. When films are grown by atomic deposition, polycrystalline structures exhibit several defects at the nano to meso scale. The initial formation of islands characterizes the growth process itself, leading to a mostly layered, nonporous structure. On the contrary, cluster-assembled films are characterized by NP-dynamics, which strongly depend on the morphology distribution of the deposited nanoparticles [1].

Computational modeling is a robust tool that provides atomistic insights into growth mechanisms. Recently, the literature has offerred numerical models to explain the complex electrical behavior of nanoparticle networks [15], as well as molecular dynamics to study the stability of nanojunctions between clusters [16,17], and the effect of the substrate in forming nano links or synapses (bridge) [13,16]. Nonetheless, to our knowledge, few works simulate the formation of nanofilms by direct cluster deposition and even fewer present a variety of morphologies used as initial seeds.

Here, we present a numerical workflow based on molecular dynamics to simulate the formation of Au nanofilms with a thickness of 25–40 nm, mimicking pulsed cluster deposition. The numerical strategy is discussed in Section 2. We expand on previous works performed on spherical, FCC nanoparticles [18,19,20,21] to AuNPs of arbitrary morphology. We consider distributions of sizes and shapes that are different and spread, aiming to better reflect the distributions of nanoparticles, as in the beam deposition experiments [1,4,22]. We contrast those experimental driven distributions with shape- and size-selected cases.

Our goal is to show whether different sizes and symmetry distributions lead to different mechanical properties in the assembled films. We analyze the influence of deposited nanoparticles distributions on film porosity and on general structural features. We examine the formation and spatial distribution of dislocations induced by the stresses generated during nanoparticle deposition, comparing systems assembled from nanoparticles exhibiting different sizes and shapes.

## 2. Methods

**Figure 1:**
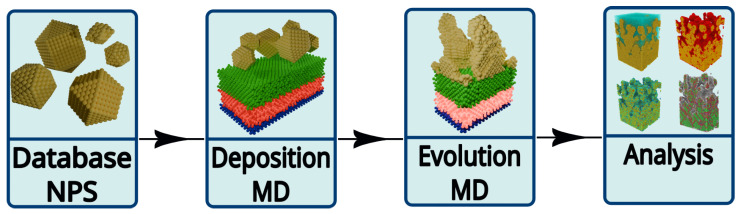
Proposed numerical workflow for the virtual synthesis of cluster-assembled films. Each box is a stage. The database is a home-built collection of Au-clusters with different sizes and shapes while the analysis tool is an original code NaMaC, combined with the tools available in Ovito. The molecular dynamics engine is LAMMPS by the scheme can be coupled with any MD-engine.

To model the virtual synthesis of cluster-assembled films, we propose the workflow depicted in Figure 1. It is based on four main steps: (i) selection of nanoparticles from a database of structures; (ii) dynamical simulation of the deposition using molecular dynamics (MD); (iii) evolution of the assembled system to achieve equilibrium through MD simulations; (iv) collection of data and analysis of the formed nanofilms.

All MD simulations were performed using the LAMMPS [23] package, with interactions mediated by the embedded atom model (EAM) [24] parametrized by Folies [25]. We used a timestep of 10 fs, in accordance with previous works [18,20] and anyhow tested on the systems considered in this work and found to not affect considerably the outcoming structural and energetic properties of the system and enabling us to achieve equilibrium on a reasonable timescale.

**Figure 2:**
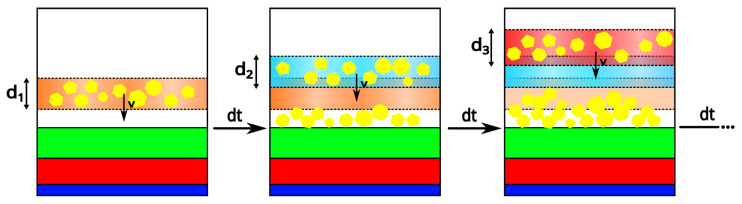
Cluster pulsed deposition model: from left to right, time evolves. The substrate has three regions: fixed layers at the bottom to replicate bulk material (blue), the thermostated layers (red), and the free-to-move regions at the top (green). The shaded region depicts the insertion box. The small yellow polygons represent the *m* AuNPs randomly placed within the insertion box. The selected AuNPs are sourced from an existing database. In the middle and right panels, differently colored boxes stand for different insertion boxes, deposited after a *dt* interval. Each insertion box possesses controllable kinetic energy, with velocity directed towards the substrate, as labeled by the black v-vector.

Stage (ii), as shown in Figure 2, mimics properly the cluster pulsed deposition: the system is divided into a substrate and an *insertion region* above it where AuNPs are initially positioned.

The substrate consists of three regions engineered to emulate a bulk surface [20,26], namely a frozen layer to represent the bulk, and a middle region of seven layers is subject to a Nosé–Hoover thermostat at room temperature. This thermalization is intended to replicate bulk absorbing the thermal energy when AuNPs reach and hit the substrate, thus maintaining a constant temperature [27]. The top region contains three Au-layers allowed to evolve freely within the NVE ensemble. The dimensions of the box are 21 × 21 nm in the xy plane, while along the *z*-axis we use a box large enough to allow for the insertion of all AuNPs and avoid the last insertion box interacting with the system-replica.

AuNPs, previously thermalized at 300 K starting from the geometrical model, are inserted within an *insertion box* positioned sufficiently above the substrate to not feel its interaction. The insertion box has a specified thickness $d_{\mathrm{insertion}}$, tunable per each insertion, spanning the entire xy-plane. The centers of mass of each AuNP are randomly distributed within this region, subject to a minimum distance threshold between newly inserted clusters and those already present. This threshold prevents excessive short-range repulsive interactions that could destabilize the system. Each nanoparticle is inserted as a LAMMPS molecule object, and, upon random insertion, their orientation is also randomized as per the default random molecule creation procedure implemented in LAMMPS.

For each box, $N_{\mathrm{tot}}$ AuNPs are inserted following the size and shape distribution shown in Table 1. We select a number of AuNPs, $N_{id}$, according to a discretization of the bimodal size distribution observed experimentally [1,4,28].

(1) $$\begin{array}{cc} N_{id}=\frac{N_{\mathrm{tot}}}{4\Delta u} & [\mathrm{Erf}\left(\frac{\Delta u+u_{0}-u_{m}}{\sqrt{2\sigma^{2}}}\right)\\ & +\mathrm{Erf}\left(\frac{\Delta u-u_{0}+u_{m}}{\sqrt{2\sigma^{2}}}\right)], \end{array}$$

where $u_{m}$ is the logarithm of the measured diameter, $d_{m}$ of the nanoparticle, Δu is the allowed size interval (still in logarithmic scale) for the AuNPs in that set, and $u_{0}$ and σ are the mean and standard deviation of the distribution, respectively. According to Equation (1), we estimate the number of AuNPs, $N_{id}$, that enables to create different sets all satisfying experimental distributions; see Table 1. The AuNP initial morphology comes from a database of geometrical built shapes, which includes icosahedra (Ih), decahedra (Dh), truncated octahedra (To), and octahedra (Oh), which are commonly observed in experiments. Those Au-seeds contain a certain number of atom $N_{a}$ and have a effective diameter $d_{id}$ calculated as their maximum pair-distance. Au-seeds are selected in such a way that their logarithmic diameter lies within Δu of the target value. We considered three sets, named after the initial morphology of the deposited AuNP as Octahedral, Icosahedral, and Mixed. While the first two sets contain AuNPs exhibiting the same geometrical shape, the Mixed combines small Ih AuNPs and large FCC-like AuNPs, in agreement with energetic consideration [29]. The total number of atoms $N_{at}$ deposited is a rough estimate of the total atoms from the experimental distribution sets, with the constraint that we fix the number of deposited AuNPs to $N_{tot}=\sum_{id}N_{id}=35$.

In addition to the three sets in Table 1, we considered other two sets that we refer to as monodispersed, and their measured average diameter is $d_{m}∼1\;\mathrm{nm}$: a set, named “Icosahedral Small” made of $N_{id}=1820$ with only Ih_55_ for $N_{at}=$ 500,500; a set, named “Octahedral Small” when $N_{id}=2500$ of Oh_40_ are in each insertion region, leading to $N_{at}=$ 500,000 atoms.

Each AuNP in the insertion box is assigned an initial velocity along the *z*-axis directed towards the substrate. The initial velocity was assigned to all the individual atoms making up the collection of nanoparticles making up each deposition step. We used an initial value of 0.8 $Å{\mathrm{ps}}^{-1}$, corresponding to a per-atom kinetic energy of 0.006 eV.

The substrate plus the inserted box is then evolved for a time period dt, tunable. During that time the free-region of the substrate and the deposited AuNPs are free to move accordingly to Newton’s equation of motion. After dt, the process of insertion and deposition is repeated until a certain amount of deposited Au. A time period of 1.5 ns was sufficient for allowing AuNPs to be deposited without clashes in the vacuum region, and to land and accommodate onto the substrate.

After the final, fifth insertion box is deposited, the system is further evolved for 15 ns. During that period, we kept track of the energy and structural properties of the system.

We repeated each individual simulation four times, each time inserting the same set of AuNPs, changing the seed of the random number generator to ensure statistical independence of the different systems.

## 3. Results and Discussion

For each of the systems outlined in the previous section, we performed four independent simulations of the same system, changing the random insertion function of LAMMPS. We describe the results as an average over the four simulations.

### 3.1. Thickness

**Figure 3:**
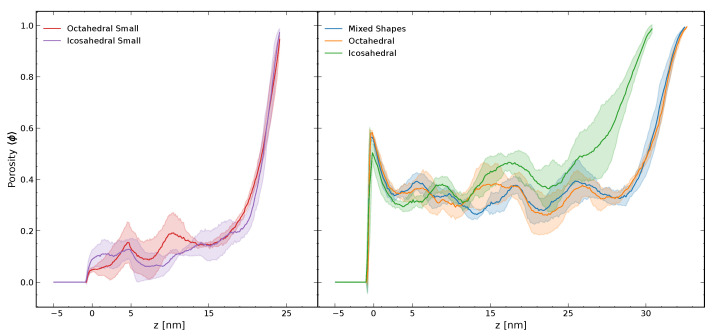
Porosity profile of considered systems deposited using experimental like distributions, and two by monodispersed small clusters. The values are averaged over four independent simulations with the same distribution of nanoparticles and varying the random seed for the insertion. The solid line is the profile averaged over the four realizations of each system. The shaded area underlies the standard deviation over the four measures.

Defining the film thickness as the maximum depth at which the porosity profile reaches unity (Figure 3), we observe a good degree of consistency among films obtained from the deposition of small clusters and those formed by experimentally distributed mixed-shape and octahedral nanoparticles. In contrast, films assembled from icosahedral clusters with an experimental size distribution are systematically thinner on average, and exhibit a narrower distribution of thickness values compared to the other two experimentally distributed systems discussed above.

This discrepancy in thickness can be explained by observing that for such systems, due to the reduced number of atoms in the larger nanoparticles, fewer atoms are inserted during the deposition simulation (Table 1). A rough calculation of the expected thickness that would be obtained by assembling the same number of atoms in an ordered crystalline structure:

(2) $$t_{cryst}=\frac{N_{at}}{N_{layer}}d_{layer},$$

where $N_{at}$ is the total number of atoms, $N_{layer}$ is the maximum number of atoms in a (111) layer, 5408 in our system and $d_{layer}$ is the distance between (111) planes, $d_{layer}=a/\sqrt{(}3)=2.35$. From Equation (2) we obtain an expected thickness of 20.7 and 25.6 nm for Icosahedral and Mixed, respectively. Octahedral is very similar to Mixed. We note that the two small sets have an expected thickness of 21.4 nm. From Figure 3, we have an estimate of the simulated thickness as the maximum thickness along the *z*-axis. We observe an average thickness of 30.5 nm for Icosahedral, about 32.5 nm for the Mixed and Octahedral sets, while small sets are just below 25 nm. We then appreciate a difference of at least 4 nm, which is about a lattice constant of difference. In general, films obtained from the deposition of individual and large AuNPs are thicker than those from epitaxy and depositing mainly icosahedral shapes at different sizes leads to thicker films.

### 3.2. Porosity

We studied the porosity in the systems using a method based on Monte Carlo integration [30], attempting to insert a certain number of probe atoms, $N_{samples}$, with a given radius, tweaked to study accessibility of the pores to different type of atoms or molecules, at random positions within the film. If the probe point is at least within a certain distance from any of the atoms in the system, including the radius of the probe atom itself, then the atom insertion is rejected, $N_{rejected}$. The porosity, ϕ, is defined as the ratio between the number of insertions over the total:

(3) $$\phi =\frac{N_{inserted}}{N_{samples}},$$

where $N_{inserted}=N_{samples}-N_{rejected}$. Besides computing the overall porosity, we also compute the porosity profile, dividing the system into slices along the z-axis and, using the same method described above to compute the porosity of each slice, this allows us to study how the porosity varies within the system along the growth direction.

The code is efficiently implemented in Python using KDtrees to improve the determination of closeness between probe and system atoms; the number of atoms has been converged until fluctuations in the porosity become negligible, and we found a good number to be 1,000,000 points for the overall porosity and 200,000 points for each slice in the porosity profile computation. For the probe atom radius, we used a value of 1.4 *Å*. The code used for the determination of the porosity was inspired by PorosityPlus [30]; we extended it to allow for an automatic determination of the cell containing the system, which in PorosityPlus was set as a fixed value to be manually determined in the input file, and to automatically perform the determination of the porosity profile.

The porosity versus thickness profile is characterized by values oscillating around the average porosity within the main body of the film, then jumping to 1 close to the surface. A study of the porosity over the studied assembled nanofilms shows an overall porosity of 0.3–0.42, see Figure 3, and around 0.15–0.2 when only small AuNPs are considered. Nanofilms assembled by smaller AuNPs are expected to show a similar behavior in the profile but with a significantly lower porosity, oscillating around 0.1–0.2. When only small AuNPs are deposited, they often undergo coalescence during their mid-flight, even forming larger films but likely with a lower geometrical order, which resulted in the formation of a less porous film. A simple explanation lies in the fact that, on a fixed timescale, smaller AuNPs can rearrange themselves more easily than large ones.

We note that the porosity profile of the system assembled using a size distribution inspired by experimental results is much closer to those obtained for the mixed-shape and purely octahedral systems [20]. This was expected, since in both cases the larger nanoparticles are predominantly octahedral (Table 1). What was less expected is that systems assembled from icosahedral nanoparticles with the same size distribution not only exhibit a markedly different porosity profile, which cannot be simply explained by the smaller numbers of total inserted atoms as was done for the thickness.

The final increase to a value close to 1 near the surface is the most characteristic feature of the shapes considered in our simulations. We observed that systems assembled using octahedra as the largest clusters generally exhibit a sharper rise to unity, whereas the presence of large icosahedral clusters results in a more gradual and less pronounced growth. This can be attributed to the fact that the final growth stage is strongly influenced by the shape of the individual nanoparticles. Since this region corresponds to the upper surface where the last deposited nanoparticles are exposed to a vacuum, we note that octahedral nanoparticles tend to form more cuboidal surface features, in contrast to the nearly spherical surface produced by icosahedra.

We observed, for a system assembled by nanoparticles with a size dispersion close to the experimental distribution, an average observed value of the porosity of 38.0±2.8%; this value is close to the average porosity of 36.8±1.8% obtained by Shpigel et al. [31] using gravimetric and beyond the gravimetric QCM-D measurements. Compared to other computational studies the value we found was higher than what was found by Benetti et al. [20] who reported a value of 27±5%; this difference can be however related to the use of a different size distribution of the primeval clusters, a different initial kinetic energy and that the system studied by Benetti and coworkers was composed of silver, which has a lower cohesive energy per atom (2.95 eV/atom) compared with the one reported for gold (3.81 eV/atom) [32], which could lead to a higher degree of deformation upon impact and thus a different overall porosity. Another work, conducted by Hanania et al. [33] reported a significantly higher value of around 90%; the main difference in their approach was the use of significantly larger nanoparticles, with an average diameter of 23 nm and that rather than an atomistic approach, impractical at those sizes, they simulated the nanoparticles as spheres, neglecting the deformation. Interestingly, from their work they observed a dependence of the porosity on the deposition process, with a resulting film being up to 10 % more porous if the deposition regime was diffusive rather than ballistic, as we considered it in the present work. For the two cases assembled by smaller nanoparticles, we observed instead an average porosity of 17.2±2.1%. One can note that the simulated average porosity values tend to be lower than the experimental values. This is expected since the porosity is inversely related to film thickness and our films are up to 40 nm less thick than experimental films.

**Figure 4:**
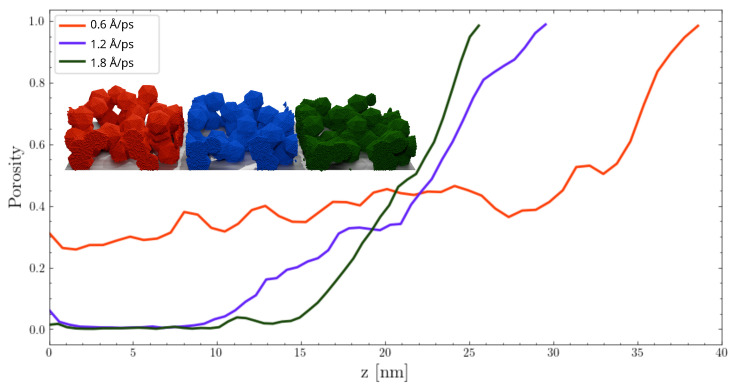
Porosity profile for the whole assembled film and rendering of the first deposited layer for three different initial velocities. This assembly was obtained with a monodispersed set of icosahedral naonparticles composed of 2057 gold atoms. The color assigned to the atoms in the render correspond to the three different velocities in the legend.

While outside the scope of this work, we tested the effect of increasing initial velocities on a system assembled by a monodispersed set of icosahedral nanoparticles individually composed of 2057 gold atoms. The velocities assigned to the individual atoms were set in three different systems to be 0.6, 1.2 and 1.8 $Å{\mathrm{ps}}^{-1}$. The resulting systems highlight the strong dependence of the porosity and thickness of the system, as well as the shape of the porosity profile, on variation of the velocity even without a change in the order of magnitude. Figure 4 reports the resulting porosity profile for the system after the deposition of the five pulses of nanoparticles and a rendering of the first pulse deposited on the substrate. It can be observed how higher velocities result in a film that is overall less porous and, with a denser packing of the same number of atoms, in a film with a smaller thickness. The result is more drastic as the velocity exceeds 1.0 $Å{\mathrm{ps}}^{-1}$, where the lower layers achieve zero porosity, resulting in a mostly filled polycrystalline bulk. Upper layers become more affected as the velocity reaches the maximum considered value of 1.8 $Å{\mathrm{ps}}^{-1}$, where the porosity remains close to zero up to around 15 nm.

### 3.3. Structural Features

We studied the structural properties of the nanofilms using an extended version of the common neighbor analysis (CNA) [34], which allows for the identification not only of typical crystal structures such as FCC, BCC and icosahedral centers, but also of features such as different types of surfaces, edges, twinning planes and other geometrical structures [35].

The original method computes the CNA signature as a pair property, and is constituted by a three-number tuple; the first is the number of common neighbors shared by the pair, the second is the number of those neighbors that are neighbors and the last is the longest uninterrupted path that can be established between the common neighbors of the pair.

Following this method we performed an analysis of the pair signatures through the different simulations on the systems taken into analysis; this allows for the identification of generic symmetries, which would be lost and not very identifiable in the large number of possible patterns that we observed in the system. The analysis was performed using the bond-based CNA implemented in Ovito [36].

We computed for each system the percentage of CNA signatures over the total number of pairs, observing how the overall largest count occurs for [4, 2, 1] signatures, associated with FCC symmetries; we also observed that the count is almost the same when we compare the system assemble by a mixed distribution of shapes and the system assembled by octahedral nanoparticles. This is due to the fact that in the system obtained by a mixture of shapes, the larger clusters are octahedral, and the variety of shapes at the smaller range of sizes is not enough to influence the system’s atomic arrangement (Table 2).

For samples made of small nanoparticles, it is possible to observe a small but not negligible number of pairs characterized by the signature [0, 0, 0], not shown in Table 2 because below 0.02%. From a visual inspection we see that the atoms forming these pairs are located on the surface, forming an almost bridge-like pair over a small vacuum, still large enough that there are no common neighbors between the atoms forming the pair. We also note that this number is higher in systems assembled by small clusters, this likely being a result of the nanoparticles losing their shape completely during deposition, resulting in a morphology more influenced by the motion of individual atoms.

The pair signature can be used to retrieve atomic properties by enumerating the signatures to which each atom contributes; such an enumeration results in a CNA pattern (CNAp) [37], which can be used to identify in which structure the atom is located. Commonly used CNA codes only identify patterns such as 12[4, 2, 1], which identifies an atom as part of an FCC crystal structure, and other common crystal structures such as BCC, HCP and two-dimensional hexagonal lattices, such as graphene. This implementation is commonly used in Ovito as a structural analysis tool [38], does not identify certain types of symmetries and completely overlooks surface features, even if ordered. This does not matter if the system under consideration is a continuous bulk; however, the identification of atoms belonging to surfaces as well as bulk is considerably useful when studying nanoparticles and nanoporous thin films [1,29,39,40] due to the high surface-to-volume ratio, such that a structural characterization based only on bulk like features will not be complete enough to study the properties of such systems.

By assigning a CNAP to the atoms, we were able to identify and uncatalog the presence of different features [35,37]. We did this by assigning an integer index to known patterns, which could then be easily compared with a table to identify the structure to which each atom belongs. With respect to the available literature providing known patterns for geometrically ideal structures, we found a few new CNAPs at necks formed between coalesced AuNPs, at the interface between the substrate and the NPs in the first insertion box, and in melted regions on the surface of the film. We observed that away from regions where significant deformations occur, only a small number of atoms are deformed to the point of changing their pattern to an unknown one, allowing us to still be able to collect worthwhile data about the symmetries and structures of the atoms. Indeed, due to the low deposition energies as in the experiments, structural rearrangements involve mostly the formation of twinning planes. Furthermore, we note that a sizable fraction of non-recognized CNAPs correspond to slight deformations of certain known patterns, deriving from distortions within the local structure, which result in places in a reduction or increase in the number of common neighbors or opening of closed chains of common neighbors or, vice versa, the closing of open chains.

**Figure 5:**
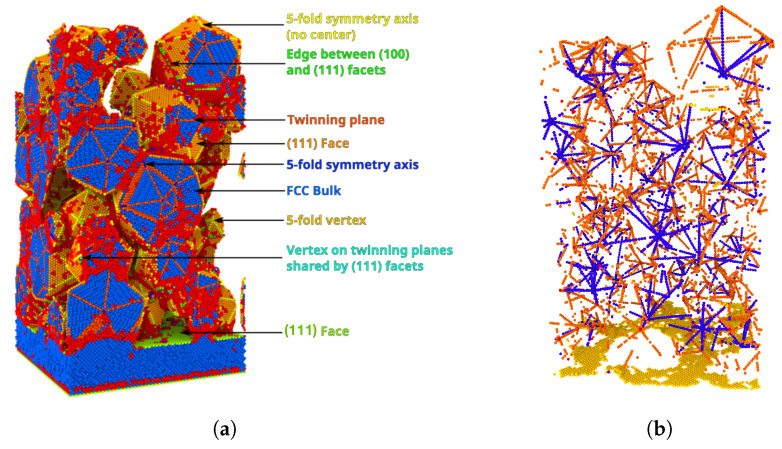
Nanofilms obtained from the deposition of icosahedral nanoparticles matching the experimental bimodal size distribution. (**a**) Structural characterization following the CNAP classification: each color represents a certain local atomic environment based on the atomic CNAP. The legend on the side associates with each recognized pattern a structural description according to the work of Jones et al. [37] (**b**) A render where only atoms with fivefold symmetries, identified by their CNA pattern, are displayed. It is possible to observe atoms belonging to internal fivefold symmetry axes (blue), edges (red), and icosahedral centers (cyan). Atoms in the upper layer of the substrate not in direct contact with deposited AuNPs are shown in yellow to indicate the substrate surface.

Interestingly, by the deposition of icosahedral nanoparticles, see Figure 5, the obtained nanofilms exhibit fivefold symmetries in the primeval AuNPs, resulting in a system where several atoms still belong to fivefold symmetries, which we can then isolate from conventional symmetries and unclassified atoms, observing how such low-energy depositions conserve to a good degree the internal symmetries of the primeval clusters; however, the internal centers of symmetries are more susceptible to small distortions and are mostly lost in the final configurations, as in Figure 5b.

### 3.4. Surface Features

Besides considering pair and atomic environment properties, we estimate the distribution of atoms located on the surface. This count is somehow related to the study of the porosity, as we can expect to find a higher number of surface atoms in regions characterized by a higher porosity. To determine the surface atoms, we used a basic threshold on the coordination number (CN), identifying as surface atoms those with a CN of 9 or lower. More effective ways of determining such a property, relying on rolling a probe particle to determine the solvent accessible surface (SAS) [41] on the surface, exist, developed in the field of bio-molecules such as proteins, though they are impractical for a system as large as the ones considered in this work.

**Figure 6:**
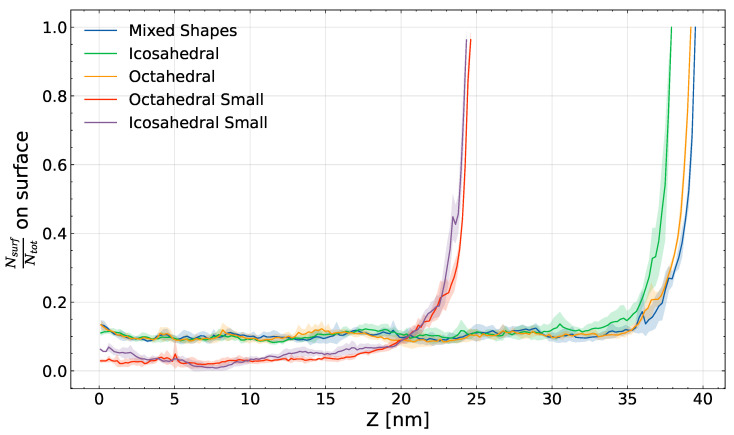
Profile along the height of the film starting from the surface of the substrate of the number of atoms identified as surface atoms ($N_{\mathrm{surf}}$) normalized by the total number of atoms at each layer ($N_{\mathrm{tot}}$) for the five sets averaged over the four independent simulations. The uncertainty over the four independent realizations of the film is reported in the shadowed region.

By binning the number of surface atoms along the growth direction with a bin size roughly equal to the lattice parameter of gold, *a*, we obtained the distribution of surface atoms with respect to the direction perpendicular to the substrate, as in Figure 6. The small oscillations of the profile in the graph depends on the fact that in OVITO, when performing a spatial binning, the user can only control the number of bins, and, over different systems with even just a small variation of thickness, the bin width can change even if only by a small value. The number of surface atoms in each bin was then normalized by the total number of atoms in each bin, resulting in a qualitatively similar curve (Figure 3 and Figure 6).

### 3.5. Dislocation Analysis

Although no mechanical study was performed in this work, a qualitative distinction in the plastic behavior of the systems can be inferred by examining the dislocation distribution, as these features correlate with plastic properties. Systems with more prevalent dislocations are generally harder, while ordered crystals are comparatively more ductile [42]. In the present systems, no external strain was applied, indicating that the dislocations arise from internal stresses due to nanoparticle impact during deposition [43]. Dislocations were identified using the Dislocation Extraction Algorithm (DXA) as implemented in OVITO [36,38], based on atomic configurations following 15 ns of equilibration. Shockley partial dislocations were found to be the most abundant type; these are characteristic of FCC metals and result from the splitting of perfect dislocations on 111 planes.

**Figure 7:**
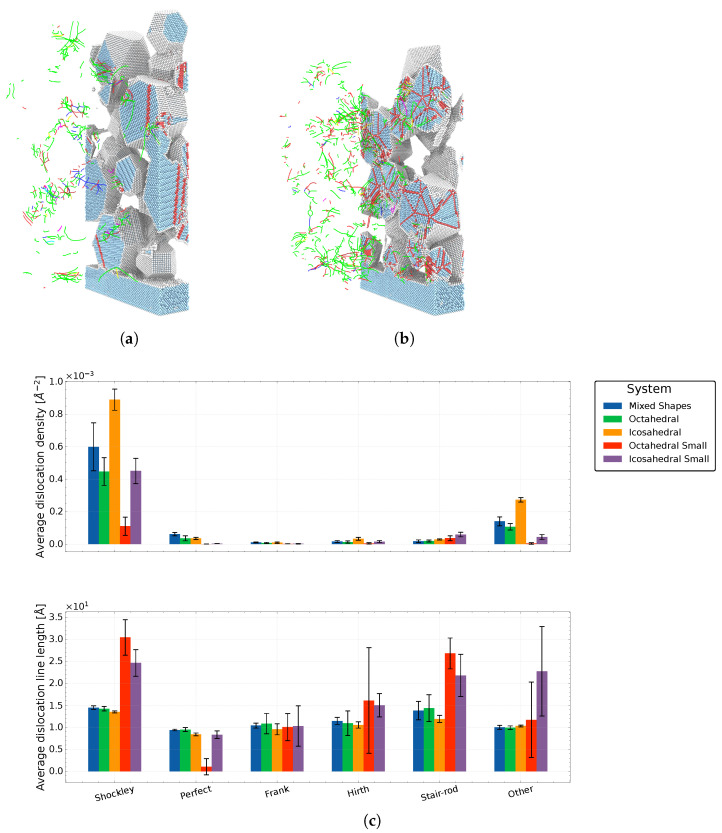
Analysis of dislocation in nanofilms: (**a**) Cross-sectional views of the dislocation line network as computed by Ovito using the DXA algorithm in systems obtained by deposition of (**a**) mixed-shape and (**b**) only icosahedral nanoparticles. Color coding: green—Shockley, blue—perfect, yellow—Hirth, magenta—stair-rod, royal blue—Frank, red—unclassified. (**c**) Density of dislocations by type across different systems with different initial nanoparticle distributions, calculated using Equation (4) and the average length of the dislocation lines for each system averaged over the different realizations of the system.

The comparison between two representative systems assembled by mixed-shape nanoparticles (Figure 7a) and icosahedral nanoparticles (Figure 7b).

Figure 7 compares two representative systems formed from mixed-shape nanoparticles (a) and icosahedral nanoparticles (b). Dislocations were visualized by slicing the film along the yz-plane, preserving partial atomic visibility. The green lines represent Shockley partials, blue lines perfect dislocations, yellow lines Hirth dislocations, magenta lines stair-rod dislocations, light blue lines Frank dislocations, and red lines denote unclassified defects.

To quantify dislocation density while accounting for porosity, the following relation was used:

(4) $$\rho =\frac{L}{V(1-\phi )},$$

where ρ is the dislocation density (in ${Å}^{-2}$), *L* is the total dislocation length, *V* is the film volume, and ϕ is the porosity. The average dislocation densities across four independent simulations for each configuration were: $1.27\times 10^{-3}\;{Å}^{-2}$ in systems assembled by deposition of purely icosahedral nanoparticles, $0.845\times 10^{-3}\;{Å}^{-2}$ in systems with mixed-shape nanoparticles, and $0.628\times 10^{-3}\;{Å}^{-2}$ in systems assembled by the deposition of purely octahedral nanoparticles. Systems assembled by smaller nanoparticles presented on average a lower density of dislocations, with an average density of $0.574\times 10^{-3}\;{Å}^{-2}$ for systems assembled by deposition of small icosahedral nanoparticles and $0.157\times 10^{-3}\;{Å}^{-2}$ for small octahedral nanoparticles as primeval clusters.

A histogram of the density of dislocations computed according to Equation (4) (Figure 7) highlights how icosahedral systems exhibit higher densities of partial and unclassified dislocations. Conversely, perfect dislocations are more prevalent in the mixed-morphology systems.

In addition, we investigated the average segment length of dislocation lines, highlighting how, while the absolute number of dislocation lines, quantified by the density, is smaller in systems assembled by small nanoparticles, their less porous structure results in dislocation lines that are in general longer and more continuous, their average length being around twice that in systems assembled by larger nanoparticles.

Further studies on the effect of what we observed are required, as dislocations can affect both structural [44,45,46], and chemical properties [47] as dislocations are also channels for the diffusion of precipitates.

## 4. Conclusions

Building on the knowledge that nanoparticles produced by cluster sources can exhibit a variety of sizes and shapes, we model and characterize nanofilms by depositing gold nanoparticles with different distributions of size and shape. We developed a numerical framework to model pulsed nanoparticle deposition and offer a numerical tool for the structural characterization of the assembly nanofilms so obtained. The nanofilm thickness is between 25 and 40 nm. We considered five nanoparticle distributions, three of which mimic the experimental size distribution, which presents two peaks around 0.6 and 9 nm. Their differences lie in their shape, where we considered only FCC-like structures, such as truncated octahedra, only icosahedra, and a mixed situation with Ih to populate the smallest-size peak and FCC for the larger sizes. We then added two distributions at small sizes, one for only FCC-like structures and one with only Ih.

Comparing the different simulations, we observed that the nanofilm porosity is poorly correlated with the shape of the deposited nanoparticles but is mainly driven by the size of the deposited seeds. The porosity of the nanofilms with a bimodal distribution in size shows a change in porosity close to the surface in films assembled by icosahedra compared to films assembled from mixed shapes and only FCC. There is a hint that the deposition of large icosahedra might lead to the formation of thicker-than-expected nanofilms. What is heavily influenced by the initial morphology of the AuNPs is the microscopic structure of the assembled films; the distribution of crystallites of different orientations throughout the system is one example of such properties, as we found, given the low velocity deposition and subsequent conservation of most of the properties of the primeval clusters, a broader, less concentrated distribution of crystalline orientations in films obtained by deposition of icosahedral nanoparticles.

A wider distribution of grains with different orientations resulted also in a larger number of possible configurations of stacking faults and other forms of grain boundaries, which in turn resulted in a larger dislocation density in films assembled by the deposition of icosahedral nanoparticles up to the larger size. The density of which is, however, instead spread in values, with uncertainties in the densities from the average over the four realizations overlapping between systems assembled by similar size distributions regardless of the shape. What we observe with more certainty is the fact that the porosity restricts the average dislocation line length and thus the characteristic bulk size.

Our work shows that the structural properties, such as thickness, roughness and surface, porosity, and dislocation density of assembly nanofilms, depend on the initial distribution of sizes and shapes of the deposited nanoparticles. Although outside of the scope of this work, such dependence can be in the future exploited to design nanofilms targeting an application, opening the way to select the best for targeting applications.

## Figures and Tables

**Table 1:** Definition of the three deposition sets named after the AuNP morphology as Octahedral, Icosahedral, and Mixed. The total number of atoms, $N_{at}$ after the five insertions is given in the second row. Per each insertion, the set is given by an AuNP identifier reporting the initial NP-shape, AuNP_*id*_, their number in the insertion, $N_{id}$, their effective diameter $d_{\mathrm{id}}$, in nm, and the corresponding measured average diameter, $d_{m}$ (nm). The line highlights the bimodal distribution of NP-sizes following experimental considerations.

		Deposited AuNP Morphology					
$N_{\mathrm{id}}$	$d_{m}$ (nm)	Octahedral		Icosahedral		Mixed	
		$N_{\mathrm{at}}$ = 592,475		$N_{\mathrm{at}}$ = 478,795		$N_{\mathrm{at}}$ = 594,095	
		AuNP_*id*_	$d_{\mathrm{id}}$	AuNP_*id*_	$d_{\mathrm{id}}$	AuNP_*id*_	$d_{\mathrm{id}}$
9	0.46	Dh_7_	0.42	Dh_7_	0.42	Dh_7_	0.42
10	0.58	To_13_	0.53	Ih_13_	0.59	Ih_13_	0.59
9	0.75	Oh_19_	0.75	Ih_55_	1.14	Ih_55_	1.14
2	4.59	To_3348_	5.06	Ih_2057_	4.39	To_3348_	5.06
3	7.57	To_8115_	7.02	Ih_8217_	7.18	To_8115_	7.02
2	12.48	To_43545_	12.71	Ih_33153_	11.61	To_43545_	12.71

**Table 2:** Percentages of CNA signatures present in a relevant number (>0.1%), at least for more situations, averaged over the four realizations of every system. Upper panel refers to CNA signature of bulk pair of atoms, while the bottom panel to surface pair of atoms. There are various defects around the FCC environment (i.e., [4, 1, 1], [4, 3, 3] on top of the common hcp stacking ([4, 2, 1]). The [4, 1, 1] is likely occurring close to necks. Atoms on a fivefold symmetry axis recognized by the [5, 5, 5], come with defects to create [5, 4, 4] and [5, 3, 3] signatures.

System	[4, 2, 1]	[4, 1, 1]	[4, 2, 2]	[6, 6, 6]	[5, 4, 4]	[5, 3, 3]	[4, 3, 3]	[5, 5, 5]
Mixed Shapes	88.647	0.195	3.493	0.028	0.698	0.011	0.807	0.047
Octahedral	88.784	0.193	3.390	0.026	0.686	0.012	0.819	0.050
Icosahedral	76.677	0.467	11.279	0.087	1.656	0.021	1.816	0.233
Octahedral Small	83.294	1.092	2.497	0.023	1.222	0.052	1.685	0.176
Icosahedral Small	81.369	0.870	4.911	0.028	1.140	0.049	1.676	0.169
**System**	**[2, 0, 0]**	**[2, 1, 1]**	**[3, 0, 0]**	**[1, 0, 0]**	**[3, 1, 1]**	**[3, 2, 2]**		
Mixed Shapes	0.194	1.601	0.132	0.007	3.755	0.318		
Octahedral	0.193	1.600	0.141	0.007	3.709	0.323		
Icosahedral	0.230	0.730	0.285	0.009	5.647	0.686		
Octahedral Small	0.990	1.035	0.629	0.193	6.095	0.849		
Icosahedral Small	1.126	1.161	0.690	0.247	5.423	0.870		

## Data Availability

The code for structural analysis, pySNOW, is available at https://github.com/nanoMLMS/pySNOW (accessed on 20 May 2026). The code used to obtain the CNA patterns is available on the GitHub page of our research group: https://github.com/nanoMLMS/CNAPatternerOvito/ (accessed on 20 May 2026), freely available. The modifier can be easily used with the graphical interface with the pro version of OVITO or with the Python interface for all users.

## Abbreviations

The following abbreviations are used in this manuscript:

MD	Molecular dynamics
EAM	Embedded atom model
CNA	Common neighbor analysis
CNAP	Common neighbor analysis pattern
NP	Nanoparticle
AuNPs	Gold nanoparticles
Ih	Icosahedron or icosahedra or icosahedral
Dh	Decahedron or decahedra or decahedral
